# SUMOylation of GTF2IRD1 Regulates Protein Partner Interactions and Ubiquitin-Mediated Degradation

**DOI:** 10.1371/journal.pone.0049283

**Published:** 2012-11-08

**Authors:** Jocelyn Widagdo, Kylie M. Taylor, Peter W. Gunning, Edna C. Hardeman, Stephen J. Palmer

**Affiliations:** 1 Neuromuscular and Regenerative Medicine Unit, School of Medical Sciences, The University of New South Wales, Sydney, New South Wales, Australia; 2 Oncology Research Unit, School of Medical Sciences, The University of New South Wales, Sydney, New South Wales, Australia; University of Minnesota, United States of America

## Abstract

*GTF2IRD1* is one of the genes implicated in Williams-Beuren syndrome, a disease caused by haploinsufficiency of certain dosage-sensitive genes within a hemizygous microdeletion of chromosome 7. GTF2IRD1 is a prime candidate for some of the major features of the disease, presumably caused by abnormally reduced abundance of this putative transcriptional repressor protein. GTF2IRD1 has been shown to interact with the E3 SUMO ligase PIASxβ, but the significance of this relationship is largely unexplored. Here, we demonstrate that GTF2IRD1 can be SUMOylated by the SUMO E2 ligase UBC9 and the level of SUMOylation is enhanced by PIASxβ. A major SUMOylation site was mapped to lysine 495 within a conserved SUMO consensus motif. SUMOylation of GTF2IRD1 alters the affinity of the protein for binding partners that contain SUMO-interacting motifs, including a novel family member of the HDAC repressor complex, ZMYM5, and PIASxβ itself. In addition, we show that GTF2IRD1 is targeted for ubiquitination and proteasomal degradation. Cross regulation by SUMOylation modulates this process, thus potentially regulating the level of GTF2IRD1 protein in the cell. These findings, concerning post-translational control over the activity and stability of GTF2IRD1, together with previous work showing how GTF2IRD1 directly regulates its own transcription levels suggest an evolutionary requirement for fine control over GTF2IRD1 activity in the cell.

## Introduction

Williams-Beuren syndrome (WBS) is a complex neurodevelopmental disorder that affects approximately 1 in 7,500–20,000 live births [1], [2]. The WBS deletion is caused by meiotic non-allelic homologous recombination between large blocks of low-copy repeats that flank the WBS critical region [3]. The hemizygous WBS deletion renders up to 28 genes haploid. Phenotypic analysis of patients with atypical deletions within the WBS critical region strongly supports the haploinsufficiency of 2 adjacent evolutionarily-related genes, *GTF2IRD1* and *GTF2I*, encoding the transcriptional regulator proteins GTF2IRD1 and TFII-I respectively, as responsible for the development of the craniofacial and prominent neurological defects [4]–[7]. The importance of GTF2IRD1 is supported by the phenotypic analysis of mice carrying mutations of the orthologous mouse gene *Gtf2ird1* [8]–[10], but a detailed understanding of the molecular function of GTF2IRD1 remains unclear.

Members of the GTF2I protein family, GTF2IRD1, TFII-I and GTF2IRD2, share highly conserved I-repeat motifs, known as repeat domains (RDs) that are unique to this family of proteins. The inter-repeat domain regions are generally less well-conserved and contain a number of predicted regulatory elements, including canonical SUMO (small ubiquitin-like modifier) motifs [11]. This motif has the consensus recognition sequence, ΨKXD/E, where Ψ represents a large hydrophobic residue and X represents any amino acid [12]. The lysine side chain forms the site of ligation for one of the three 11-kDa SUMO peptides, SUMO1, 2 and 3.

Similar to ubiquitination, the SUMO conjugation pathway involves a cascade of enzymatic processes catalysed by the E1 SUMO-activating enzyme, the E2-conjugating enzyme (UBC9), and an E3 ligase, which is involved in substrate target selection and promotes the transfer of SUMO from UBC9 to the target protein. The protein inhibitor of activated STAT (PIAS) family of proteins act as E3 ligases and are the mammalian homologs of the yeast SIZ proteins [13]. SUMOylation has emerged as a powerful regulator of chromatin, transcription and signal transduction, with the majority of SUMO substrates being nuclear localized proteins [14]. The functional consequences of SUMOylation on the ligated substrate include alterations in subcellular and sub-nuclear localization, transcriptional mechanisms, as well as protein stability via a cross-regulation with the ubiquitin-proteasome degradation pathway [15]. At the molecular level, these functional outcomes are often achieved through changes in the protein-protein interactions of SUMOylated proteins, which may involve changes in protein conformation, inhibition of an existing binding site or creation of a new binding module for SUMO-interacting motif (SIM)-containing proteins [16]. SIMs have been identified in elements of the SUMOylation machinery including the PIAS family [17], proteins involved in the SUMO-dependent repression of gene transcription [14] and more recently, members of the ubiquitin machinery [18].

Previous reports have indicated that GTF2IRD1 and TFII-I are capable of directly binding to the E3 ligase PIASxβ [19] and TFII-I and GTF2IRD2 have been identified in broad proteomic screens as SUMO substrates [20]–[22]. Although protein sequence analysis indicates potential SUMOylation sites in GTF2IRD1 [23], there is no direct evidence to support such a post-translational modification or what the consequences of SUMOylation might be.

Here we demonstrate that GTF2IRD1 is targeted by the endogenous SUMOylation machinery in HEK293 cells at Lys-495 of the human protein, which has a significant impact on the affinity of GTF2IRD1 for 2 SIM-containing proteins; the E3 ligase itself, PIASxβ and a novel interacting partner, the putative histone deacetylase (HDAC)-associated protein, ZMYM5. Furthermore, we demonstrate that GTF2IRD1 is subject to ubiquitination and proteasomal degradation. Positive cross-modulation between SUMOylation and ubiquitination potentially regulates overall GTF2IRD1 protein stability. This report provides the first molecular insight into the post-translational regulatory mechanisms of GTF2IRD1.

## Materials and Methods

### Plasmid constructs

**Figure 1:**
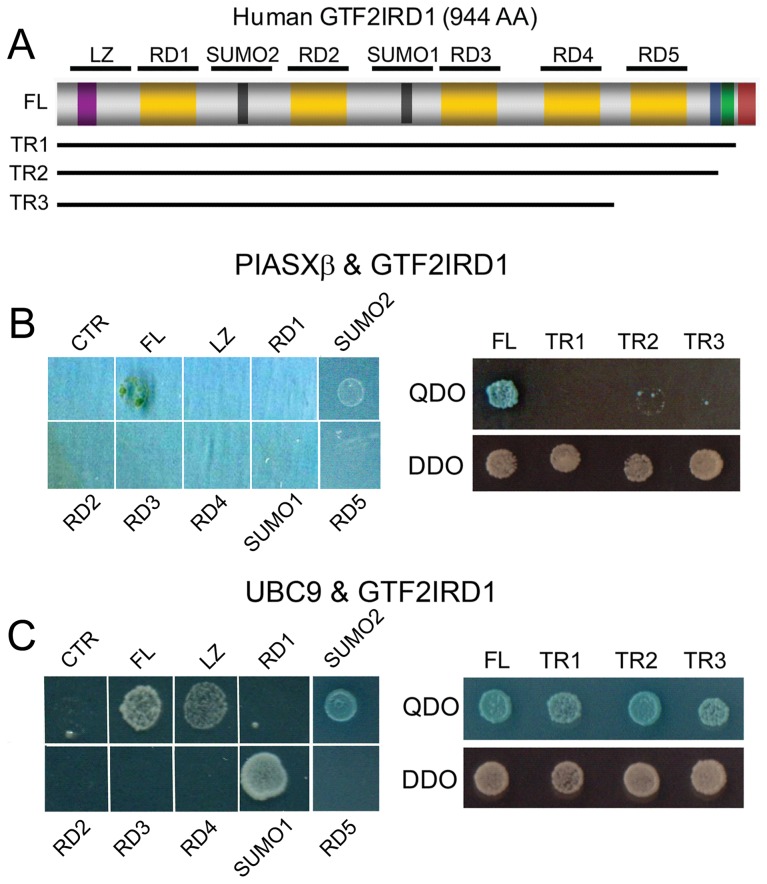
GTF2IRD1 binds to the E3 SUMO ligase PIASxβ and the E2 SUMO conjugating enzyme UBC9 in yeast assays. (A) Schematic diagram of human GTF2IRD1 protein structure and its various domains. The peptide regions used to map protein binding sites are indicated by the annotated thick lines above and the C-terminal truncations (TR1–3) are indicated below. The domains (from left to right) include a leucine zipper (LZ, purple), the repeat domains (RD1–5, gold), SUMO attachment sites (SUMO1 & 2, black), nuclear localization signal (blue), polyserine tract (green) and the conserved C-terminal domain (maroon). (B) Yeast 2-hybrid assays confirming and mapping the GTF2IRD1 interaction with PIASxβ. Double transformations were performed using pGADT7-PIASxβ plus the control empty pGBKT7 bait plasmid (CTR), pGBKT7-GTF2IRD1 full-length (FL), or the peptide regions indicated in A or the C-terminal truncation fragments (TR1–3). Positive interactions are indicated by survival on QDO plates and blue α-galactosidase staining. Survival on DDO plates was tested for all double transformants to ensure the presence of bait and prey plasmids in the yeast host (C) A similar assay testing the binding of GTF2IRD1 to UBC9 and mapping the interaction sites.

For yeast 2-hybrid assays, bait and prey sequences were cloned into pGBKT7 (containing the GAL4 DNA-binding domain) and pGADT7 (containing the GAL4 activation domain) vectors, respectively (Matchmaker system, Clontech). pGBKT7 plasmids encoding the full-length human GTF2IRD1 1α1 isoform [24], specific domains (LZ, RD1, RD2, RD3, RD4, RD5, SUMO1, SUMO2), and the C-terminal truncations of GTF2IRD1 (TR1: aa 1–932, TR2: aa 1–909, TR3: aa 1–787; Fig. 1A) were generated by PCR amplification using pcDNA-GTF2IRD1 (see below) as a template and the primers listed in Table S1. pGADT7-PIASxβ and pGADT7-UBC9 were generated by PCR amplification of the *Pias2* and *Ube2i* open reading frames (ORFs) from mouse cDNA using PIASxF-R and UBC9F-R primers (Table S1). pGADT7 constructs containing C-terminal truncations of ZMYM5 were generated using the isolated *Zmym5* prey plasmid as a template in the PCR amplification.

The mammalian expression plasmid, pcDNA-GTF2IRD1, was generated by PCR amplification of the human GTF2IRD1 ORF using HMDCDSF and HMDCDSR primers (Table S1) and ligation into the pcDNA3.1 vector (Invitrogen). The amino-terminal epitope-tagged plasmid, pMyc-GTF2IRD1, was made by insertion of a synthesized oligonucleotide linker fragment encoding the Myc epitope into pcDNA-GTF2IRD1 and pEGFP-GTF2IRD1 was made by excision of the *GTF2IRD1* ORF from pcDNA-GTF2IRD1 and ligation into pEGFP-C3 (Clontech). Plasmids encoding the K495R and K271R mutant forms of the GTF2IRD1 protein were made by site-directed mutagenesis using the standard splicing by overlap extension (SOE) PCR-based mutagenesis protocol [25] with pcDNA-GTF2IRD1 as a template. The combined 2KR (K495R/K271R) was generated by restriction enzyme excision of a fragment encoding the region around K495 in pcDNA-K271R plasmid and replacement with the equivalent fragment from pcDNA-K495R. pEGFP-2KR was generated by excision of the double mutant GTF2IRD1 fragment from pcDNA-2KR and insertion into pEGFP-C3. HA-SUMO (pMT3-HA-SUMO1) containing the mature form of SUMO1 with pre-exposed C-terminal double glycine and tagged with HA-epitope has been described previously [26]. pcDNA-UBC9 and pEGFP-PIASxβ generated using the mouse *Ube2i* and *Pias2* ORFs described earlier, ligated into pcDNA3.1 and pEGFP-C1 (Clontech) respectively. pEGFP-ZMYM5 was generated by PCR amplification of the *Zmym5* ORF from mouse cDNA using ZMYM5F-R primers. FUGW-Myc-Ubiquitin and pRK5-HA-Ubiquitin were gifts from Dr. Victor Anggono.

### Yeast 2-hybrid assay and library screening

All yeast 2-hybrid strategies were based on the Matchmaker system (Clontech). Small scale yeast transformation in *S. cerevisiae* strain AH109 to test candidate interactions was performed as described elsewhere [27]. Selection of double transformants was achieved by growth on double dropout (DDO) Trp/Leu-deficient, synthetic defined (SD), selection agar plates. Resistant colonies were re-spotted onto quadruple dropout (QDO) Trp/Leu/His/Ade-deficient SD plates containing x-α-galactosidase (Clontech) to test for evidence of interaction by activation of the *HIS3*, *ADE2* and *MEL1* selection markers.

The universal, normalized, mouse cDNA yeast 2-hybrid library (Cat. No. 630483, Mate & Plate, Clontech) was screened according to the manufacturer's instructions using pGBKT7-GTF2IRD1 as the bait plasmid. Resistant α-galactosidase-positive colonies were isolated for plasmid rescue and insert sequencing according to the recommended protocol (Clontech).

### Cell lines and transient transfections

COS-7 and HEK293 cells were grown in Dulbecco's modified Eagle's medium, supplemented with 10% foetal bovine serum, L-glutamine (2 mM), penicillin and streptomycin at 37°C in a humidified 5% CO2 incubator. COS-7 cells were transfected using GeneJuice (Merck) according to the manufacturer's protocol, while HEK293 cells were transfected using the standard calcium phosphate precipitation method. For proteasomal degradation studies, cycloheximide was added to the culture medium 24 h after transfection to a final concentration of 100 µg/ml. The proteasomal degradation inhibitor, MG132, was added to a final concentration of 20 µM 30 min prior to the addition of cycloheximide.

### Protein extraction, immunoprecipitation and Western blot analysis

For total cell extracts, cells were washed in PBS and lysed in Laemmli buffer. For nuclear extracts, HEK293 cells were lysed in Buffer A (10 mM HEPES, 1.5 mM MgCl_2_, 10 mM KCl, 0.5 mM DTT, 0.05% NP40, pH 7.9) for 10 min on ice then centrifuged at 1000×*g* for 10 min. The pellet was resuspended in Buffer B (5 mM HEPES, 1.5 mM MgCl_2_, 0.3 mM NaCl, 0.2 mM EDTA, 0.5 mM DTT, 26% glycerol, pH 7.9), incubated on ice for 30 min, centrifuged at 24000×*g* for 20 min at 4°C, and supernatant was recovered as the nuclear fraction. For immunoprecipitation, cells were lysed in either RIPA buffer (SUMOylation and ubiquitination assays) or PBS containing 0.1% v/v Triton-X (for GTF2IRD1-ZMYM5 co-immunoprecipitation assays) as previously described [28]. For ubiquitination and endogenous SUMOylation studies, N-ethylmaleimide (NEM) was added to the extraction buffer to a final concentration of 20 mM. For immunoprecipitation, primary antibodies including: anti-GTF2IRD1 (WBSCR11 [M-19] Santa-Cruz), anti-HA (Y-11, Santa Cruz) or anti-GFP (ab290, Abcam) were coupled to beads for 1 h at 4°C. Pre-cleared lysates were incubated with the antibody-bound beads at 4°C overnight. Beads were washed in lysis buffer 3 times by re-suspension, centrifugation at 845×*g* and aspiration of fluid, followed by elution of proteins by boiling in 2× Laemmli sample buffer. Proteins were resolved by SDS-PAGE and transferred to PVDF membrane by Western blotting. Western blot probe antibodies included anti-GTF2IRD1 (as above), anti-HA (as above), anti-Myc (9E10 clone, Sigma), anti-GFP (FL sc-8334, Santa Cruz), anti-SUMO1 (S5446, Sigma) and anti-α-tubulin (DM1A, Sigma). Densitometry was performed by image capture using an ImageQuant LAS 4000 (GE Healthcare) and analysis of band intensity using ImageJ software (http://imagej.nih.gov/ij). Statistical analysis of data was performed using Prism software (GraphPad).

### Immunofluorescence

Cells were cultured on sterile glass coverslips seated in a 12-well plate and transfected as described above. Coverslips were removed 24 h post-transfection, washed once in PBS prior to fixation of the cells in ice-cold methanol for 10 min at −20°C. Myc-GTF2IRD1 was detected by standard immunofluorescence methods using blocking buffer (10% BSA in PBS) for 1 h at room temperature, incubation of the primary antibody (anti-Myc 9E10, Sigma), a fluorescent secondary antibody (goat anti-mouse Alexa Fluor 555, Invitrogen) and mounting in Vectashield (Vector Laboratories) containing 1.5 µg/ml DAPI. Images were acquired using a Zeiss AxioCam MRc mounted on an AxioSkop40 epifluorescence microscope.

## Results

### GTF2IRD1 can interact with UBC9 and PIASxβ

To investigate the possibility of GTF2IRD1 SUMOylation at the predicted SUMOylation sites (Fig. 1A), we first characterized potential interactions between GTF2IRD1 and two key proteins involved in the SUMO pathway, UBC9 and PIASxβ. A candidate yeast 2-hybrid assay performed by co-transformation of pGBKT7-GTF2IRD1 and pGADT7-PIASxβ confirmed the previously reported yeast 2-hybrid interaction between full-length human GTF2IRD1 and the E3 SUMO ligase, PIASxβ [19], as shown by activation of the *HIS3*, *ADE2* and *MEL1* selection markers during growth on QDO plates (Fig. 1B). Similarly, co-transformation of pGBKT7-GTF2IRD1 and pGADT7-UBC9 plasmids into yeast revealed a positive interaction between GTF2IRD1 and this SUMO E2 conjugating enzyme (Fig. 1C). A negative control co-transformation with pGBKT7-GTF2IRD1 and empty pGADT7 confirmed the inability of GTF2IRD1 to auto-activate the selectable markers or to interact with the GAL4 activation domain (data not shown). Conversely, co-transformation with pGBKT7 and pGADT7-PIASxβ or pGADT7-UBC9 resulted in no growth on QDO plates confirming the absence of non-specific binding of PIASxβ and UBC9 to the GAL4 DNA binding domain (Fig. 1B and C).

To map the regions within GTF2IRD1 that are responsible for binding to PIASxβ and UBC9, the full-length human protein was divided into regions of strong evolutionary conservation. These include the leucine zipper region near the N-terminus (LZ), the five repeat domains (RD1–5) and two SUMO consensus motif-containing regions (SUMO1 and SUMO2) (Fig. 1A). CDNA sequences corresponding to these regions, encoding peptides 88 amino-acid in length, were inserted into the pGBKT7 bait plasmid. Each of these plasmids were confirmed negative for auto-activation of the yeast selectable marker by co-transforming with pGADT7 plasmid. However, a bait plasmid encoding the carboxy-terminal domain of GTF2IRD1 fused to the GAL4 DNA binding domain auto-activated the selectable markers in the presence of pGADT7 plasmid. Thus, a series of GTF2IRD1 C-terminal truncations were generated as an alternative means of testing the interactive capabilities of this region (TR1–3 [Fig. 1A]).

Analysis using these constructs indicated that PIASxβ interacts with the extreme C-terminal end of GTF2IRD1, since survival on QDO plates was abolished with all of the TR series including TR1, in which only the last 27 amino acids are missing from the protein (Fig. 1B). Similar tests using pGBKT7-UBC9 revealed that UBC9 interacts with GTF2IRD1 via the SUMO1 and SUMO2 regions (Fig. 1C), which was expected according to its recognized properties for direct binding to SUMO motifs [12]. The weak colony growth observed with co-transformed UBC9 and LZ region (Fig. 1C) was unexpected since this region is known to mediate dimerization of GTF2IRD1 [29] and no SUMO consensus motifs are found within this domain. However, an RKDE sequence is present, which may be sufficiently close to the consensus to permit weak binding.

### GTF2IRD1 is SUMOylated

**Figure 2:**
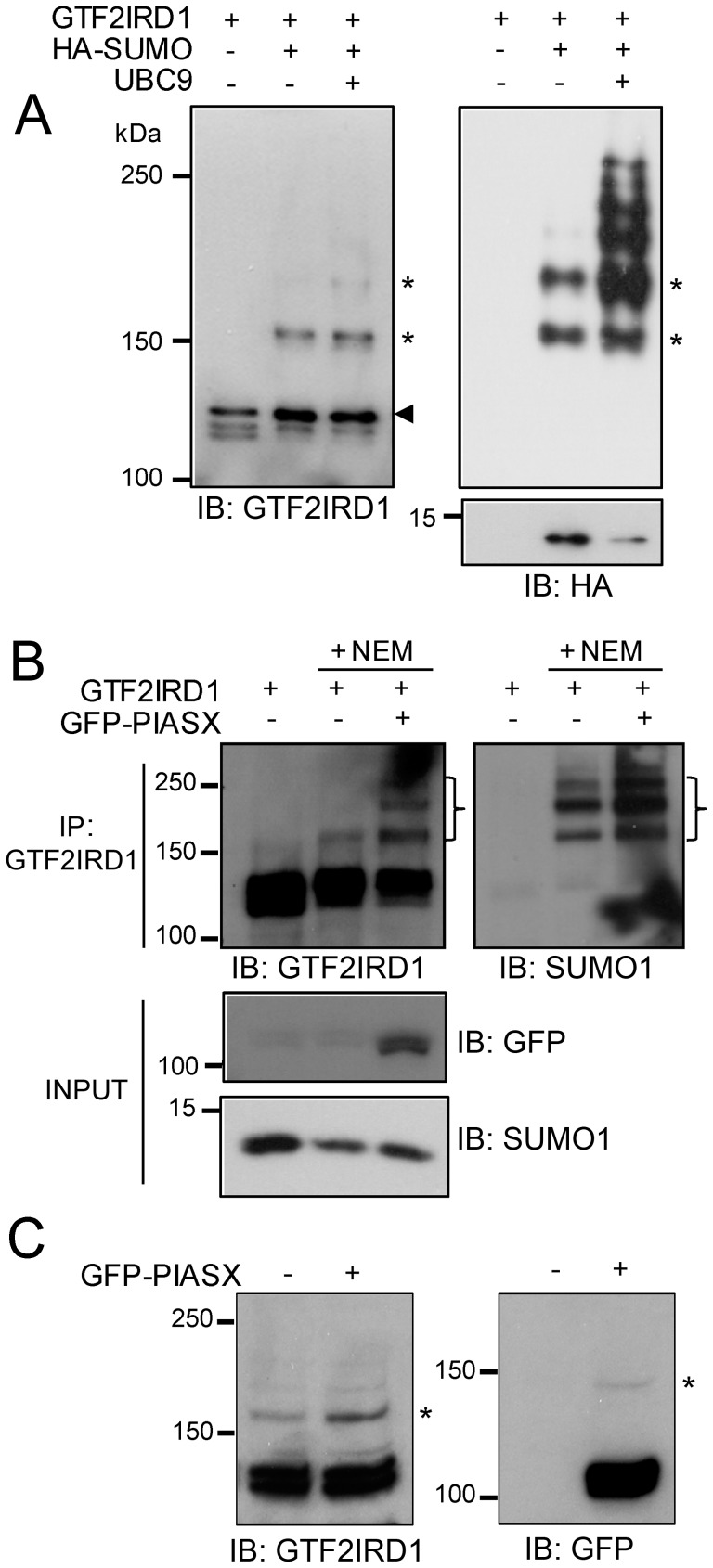
SUMOylation of GTF2IRD1. (A) Western blots showing total protein extracts from COS-7 cells transfected with pcDNA-GTF2IRD1, pMT3-HA-SUMO1 and pcDNA-UBC9 and immunoblotted (IB) with anti-GTF2IRD1 and anti-HA tag antibody. The arrowhead indicates the GTF2IRD1 cognate band and the asterisks correspond to the higher molecular weight SUMOylated forms of GTF2IRD1. The unconjugated HA-SUMO peptide of approximately 11 kDa is shown in the bottom panel. (B) Western blots showing protein extracts from HEK293 cells transfected with pcDNA-GTF2IRD1 and pEGFP-PIASxβ lysed in the absence or presence of NEM and subjected to immunoprecipitation (IP) with anti-GTF2IRD1 antibody and immunoblot analysis using anti-GTF2IRD1 and anti-SUMO1 specific antibodies. A ladder of bands (}) indicates SUMOylation of GTF2IRD1 by endogenous SUMO1. Unconjugated endogenous SUMO1 is detectable in the whole cell extract (INPUT). (C) Western blots showing nuclear fractions from HEK293 cells with or without transfected pEGFP-PIASxβ. The asterisks indicate SUMOylated forms of endogenous GTF2IRD1 (left panel) and exogenous GFP-PIASxβ (right panel).

Having established the interactions between GTF2IRD1 and the SUMO E2 and E3 ligases, we then examined if GTF2IRD1 can be SUMOylated in mammalian cell lines. COS-7 cells were transiently transfected with plasmids encoding full-length human GTF2IRD1 (pcDNA-GTF2IRD1) and HA-tagged SUMO1 (pMT3-HA-SUMO1), with or without the UBC9-expression construct (pcDNA-UBC9). Co-expression of GTF2IRD1 and the HA-SUMO resulted in the robust detection of a novel high molecular weight band slightly above 150 kDa and a weaker band at approximately 180 kDa, in addition to the lower cognate band of GTF2IRD1, which runs at approximately 120 kDa (Fig. 2A). These higher molecular weight bands are consistent with the covalent modification of GTF2IRD1 with SUMO moieties. To test this hypothesis, the membrane was stripped and re-probed with the anti-HA antibody, which identified the two high molecular weight bands and some additional higher molecular weight bands, but not the 120 kDa unmodified GTF2IRD1 (Fig. 2A). Overexpression of UBC9 increased the amount of SUMO-conjugated protein detected in the cell extracts, consistent with GTF2IRD1 being a novel substrate of the UBC9 enzyme.

We then sought to find out whether GTF2IRD1 can be SUMOylated by the endogenous cellular machinery. Unlike COS-7 cells, HEK293 cells are known to contain a relatively large pool of SUMO1 protein [30] and thus were used for these experiments. HEK293 cells were transfected with pcDNA-GTF2IRD1 and lysed in the presence or absence of N-ethylmaleimide (NEM), the SUMO-isopeptidase inhibitor. Western blot analysis of immunoprecipitated GTF2IRD1 proteins revealed the presence of a higher molecular weight band in the presence, but not in the absence of NEM (Fig. 2B). The identity of the higher molecular weight bands as SUMO conjugates of GTF2IRD1 was confirmed by re-probing the membrane with an anti-SUMO1 specific antibody (Fig. 2B). In this experiment, co-transfection of plasmids encoding GTF2IRD1 and the GFP-tagged PIASxβ led to enhanced levels of GTF2IRD1 SUMOylation (Fig. 2B), supporting the functional role of PIASxβ as a SUMO E3 ligase that enhances the targeting of GTF2IRD1 for SUMOylation.

Evidence for SUMOylation of endogenous GTF2IRD1 was examined by Western blot analysis of nuclear extracts made from HEK293 cells in the presence of NEM and immunoblotting with anti-GTF2IRD1 antibodies (Fig. 2C). In addition to the cognate GTF2IRD1 band at approximately 120kDa, higher molecular weight bands corresponding to SUMOylated GTF2IRD1 species were detected in the immunoblot, which were enhanced in the presence of overexpressed GFP-PIASxβ (Fig. 2C). To confirm the presence of GFP-PIASxβ, the blot was stripped and reprobed with anti-GFP antibody, revealing the main PIASxβ band and a higher molecular band consistent with SUMOylated PIASxβ as expected [31] (Fig. 2C).

### SUMOylation site mapped to lysine 495 of GTF2IRD1

**Figure 3:**
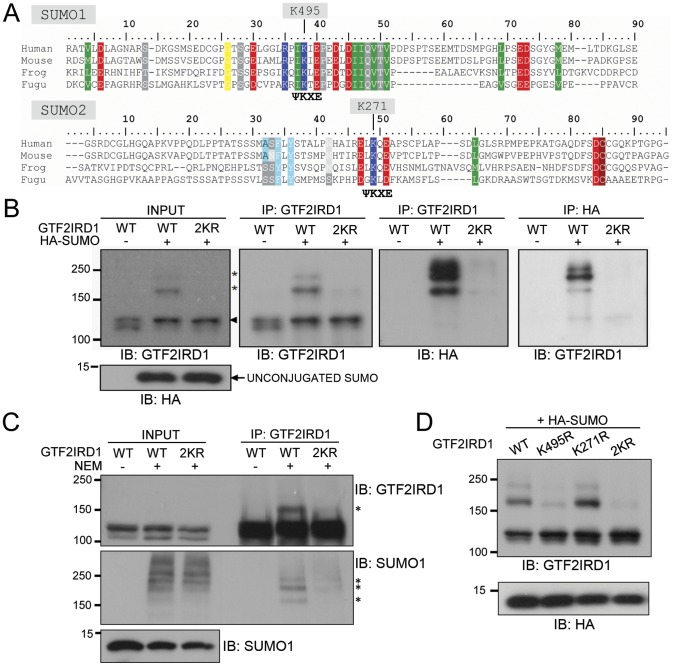
GTF2IRD1 is SUMOylated primarily on lysine 495. (A) ClustalW alignments of the SUMO1 and SUMO2 regions of GTF2IRD1 in human, mouse, frog and fugu showing conservation of amino acid sequence (identity/similarity is highlighted) and the positions of the SUMO consensus motifs (ΨKXE). (B) Western blots of protein extracts from COS-7 cells transfected with plasmids encoding wild type GTF2IRD1 (WT) or the double mutant K271R/K495R (2KR) and HA-SUMO and then subjected to immunoprecipitation (IP) with anti-GTF2IRD1 and anti-HA specific antibodies followed by immunoblot analysis using the indicated antibodies. The arrowhead and asterisks indicate the non-SUMOylated and SUMOylated GTF2IRD1 bands, respectively. (C) Western blots of protein extracts from HEK293 cells transfected with pcDNA-GTF2IRD1 (WT) or the 2KR mutant version (2KR) lysed in the presence or absence of NEM. Whole cell extract (INPUT) or proteins immunoprecipitated using the anti-GTF2IRD1 antibody were probed with anti-GTF2IRD1 or anti-SUMO1 specific antibodies. Asterisks indicate GTF2IRD1 protein modified by endogenous SUMO1 attachment. (D) Western blot analysis of total protein extracts from COS-7 cells transfected with pMT3-HA-SUMO1 and pcDNA-GTF2IRD1 (WT), the equivalent plasmids that encode the single lysine mutants (K495R and K271R) or the double mutant (2KR), probed with anti-GTF2IRD1 antibody.

To determine the sites of SUMO ligation, plasmids encoding GTF2IRD1 with lysine to arginine mutations of Lys-495 and Lys-271 were prepared, either individually (K495R and K271R) or in combination (2KR), by site-directed mutagenesis (Fig. 3A). Plasmids encoding HA-SUMO and wild type GTF2IRD1 were co-transfected into COS-7 cells, followed by reciprocal co-immunoprecipitation using anti-GTF2IRD1 and anti-HA antibodies, which resulted in detection of the higher molecular weight SUMOylated GTF2IRD1 species, as seen previously, but when the co-transfections were performed with the 2KR mutant form of GTF2IRD1, the higher molecular weight SUMOylated bands were severely reduced (Fig. 3B). A similar result was obtained using transfection of GTF2IRD1 and 2KR into HEK293 cells, followed by detection of SUMOylation by the endogenous SUMO1 (Fig. 3C).

To determine the relative efficiency of SUMO ligation to K495 and K271, constructs encoding GTF2IRD1 with the individual mutations were co-transfected into COS-7 cells with HA-SUMO. This experiment revealed that the level of SUMOylation found on the K271R mutant was comparable to that of the wild type GTF2IRD1 protein (Fig. 3D). In contrast, SUMOylation of the K495R mutant protein was reduced to a level similar to the 2KR protein (Fig. 3D), indicating that virtually all of the SUMOylation of GTF2IRD1 occurs at K495.

### SUMOylation of GTF2IRD1 modulates its interaction with two SIM-containing proteins: PIASxβ and ZMYM5

**Figure 4:**
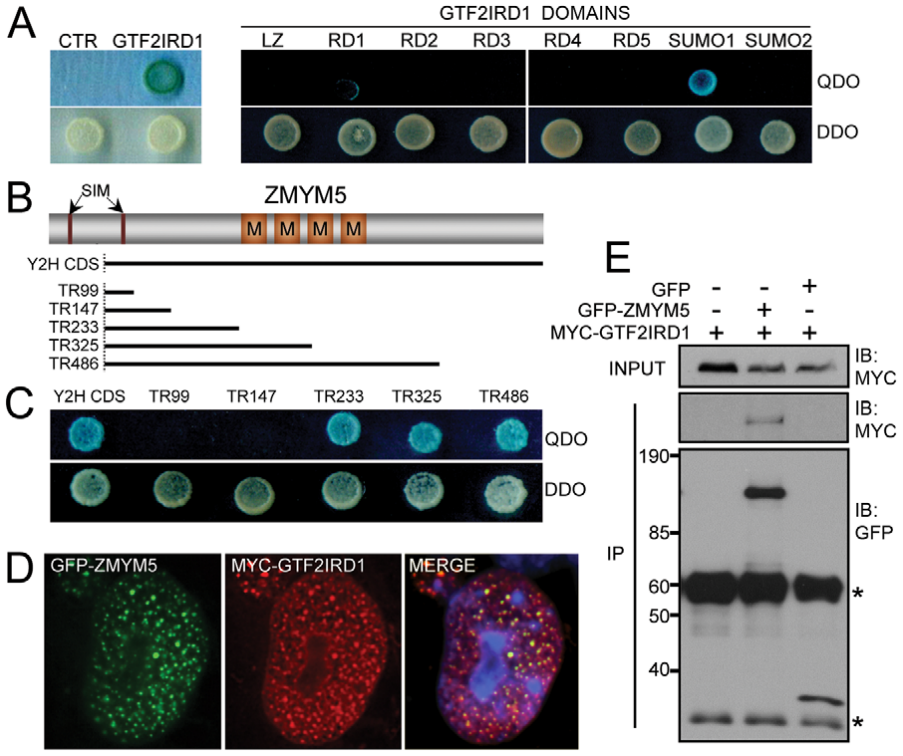
Characterization of a novel interaction between GTF2IRD1 and ZMYM5. (A) Yeast 2-hybrid assays showing relative survival and α-galactosidase activation of yeast colonies on QDO and DDO plates. The yeast cells were co-transformed with pGADT7-ZMYM5 and a variety of bait plasmids including an empty pGBKT7 vector control (CTR), full-length GTF2IRD1 and the individual domains of GTF2IRD1. (B) Schematic diagram of ZMYM5 showing the position of 2 SUMO-interacting motifs (SIMs) and the MYM-type zinc finger domains (M). The black bars below indicate the extent of the ORF found in the ZMYM5 clone isolated in the original yeast 2-hybrid screen (Y2H CDS) and the truncated forms (TR99–TR486) generated to map the interaction domain. (C) Yeast 2-hybrid analysis of GTF2IRD1 interaction domain in ZMYM5 using the truncation series (TR99–TR486). (D) Immunofluorescence analysis of COS-7 cells transfected with pEGFP-ZMYM5 and pMyc-GTF2IRD1 showing extensive colocalization in the nucleus as illustrated by the overlay of GFP, anti-Myc immunofluorescence and DAPI (MERGE). (E) Western blot analysis of protein extracts from HEK293 cells co-transfected with pEGFP or pEGFP-ZMYM5 and pMyc-GTF2IRD1 plasmids. Whole cell extracts (INPUT) or proteins immunoprecipitated using anti-GFP antibody (IP) were immunoblotted (IB) with anti-Myc and anti-GFP antibodies. GFP-ZMYM5 and GFP were detected at approximately 100 kDa and 25 kDa respectively. Asterisks indicate IgG heavy and light chains.

Since the functional consequences of SUMOylation are often mediated by changes in the protein-protein interactions of the ligated substrate, we wanted to explore the impact of SUMOylation on GTF2IRD1's protein binding capabilities. As part of an effort to determine novel protein partners of GTF2IRD1 (Widagdo et al., manuscript in preparation), a novel interaction with the protein ZMYM5 [32], [33] was identified in a yeast 2-hybrid library screen using the full-length human GTF2IRD1 protein as a bait. A library colony surviving QDO selection was found to contain an amino-terminal truncated ZMYM5 lacking the first 62 amino acids. Re-transformation of the rescued ZMYM5 prey plasmid into AH109 yeast in conjunction with either the bait plasmid or an empty pGBKT7 control vector confirmed the GTF2IRD1-ZMYM5 interaction (Fig. 4A).

To map the binding site of ZMYM5 within GTF2IRD1, bait plasmids containing each of the specific domains of GTF2IRD1 (Fig. 1A) were co-transformed into yeast with the isolated pGADT7-ZMYM5 plasmid. This experiment revealed that the binding site was located within the SUMO1 region, which also contains the principle site of SUMO ligation at K495 (Fig. 4A).

Conversely, to identify the region in ZMYM5 that is responsible for interaction with GTF2IRD1, serial truncations of the isolated yeast 2-hybrid clone were made (Fig. 4B). Yeast 2-hybrid assays using these plasmids, in combination with the full-length GTF2IRD1, revealed that the binding region in ZMYM5 mapped to amino acid range 148–233, which precedes the ‘MYM’-type zinc finger repeats characteristic of the ZMYM proteins (Fig. 4C).

To examine the sub-cellular localization of ZMYM5 and its potential interaction with GTF2IRD1 in mammalian cells, the full-length *Zmym5* ORF was cloned into a GFP-tagged expression plasmid. Immunofluorescence analysis of COS-7 cells transfected with pEGFP-ZMYM5 and pMyc-GTF2IRD1 revealed that the GFP-ZMYM5 protein was distributed throughout the nucleus in a punctate pattern that overlapped extensively with foci of Myc-GTF2IRD1 (Fig. 4D).

To confirm the interaction between ZMYM5 and GTF2IRD1 in mammalian cells, HEK293 cells were co-transfected with pMyc-GTF2IRD1 and pEGFP-ZMYM5 or the pEGFP vector control. Western blot analysis of cell lysates immunoprecipitated with anti-GFP antibody showed co-immunoprecipitation of Myc-GTF2IRD1 in the GFP-ZMYM5 immunocomplex (Fig. 4E), thus supporting the yeast 2-hybrid results and the sub-cellular localization data. Myc-GTF2IRD1 was undetectable in the negative control immunoprecipitates containing Myc-GTF2IRD1 alone or Myc-GTF2IRD1 with EGFP (Fig. 4E).

**Figure 5:**
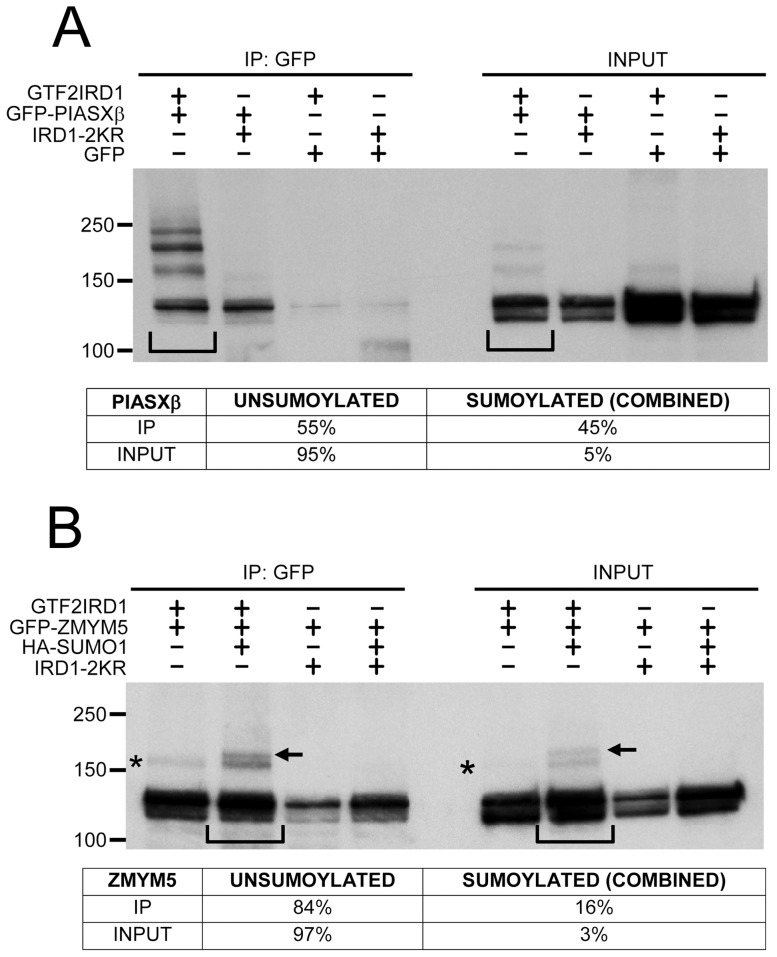
SUMOylation of GTF2IRD1 increases interaction with PIASxβ and ZMYM5, a novel HDAC-interacting protein. (A) Western blot analysis of protein extracts from HEK293 cells transfected with plasmids encoding GTF2IRD1, GTF2IRD1-2KR (IRD1-2KR), GFP-PIASxβ or EGFP control. Whole cell extracts (INPUT), and proteins immunoprecipitated (IP) using the anti-GFP antibody, were immunoblotted with anti-GTF2IRD1 antibody. The increased relative proportions of SUMOylated GTF2IRD1 (ladder of bands running at >150 kDa) over the non-SUMOylated GTF2IRD1 (running at approximately 120 kDa) in the IP lanes relative to the input lanes (compare the lanes indicated by the square brackets) indicate a binding preference of GFP-PIASxβ for the SUMO-conjugated forms of GTF2IRD1. Quantitative densitometric analysis of this comparison is shown in the table below. The image shown is a representative example of 2 separate experiments with similar results. (B) Similar co-immunoprecipitation analysis of GFP-ZMYM5 and the non-SUMOylated/SUMOylated versions of GTF2IRD1 in HEK293 cells co-transfected with pMT3-HA-SUMO1. SUMOylated GTF2IRD1 bands due to the exogenously-derived HA-SUMO1 or endogenous SUMO are indicated by the arrows and asterisks, respectively. Densitometry of the band intensities in the lanes marked with square brackets is shown in the table below and indicates that GFP-ZMYM5 also has a preference for binding to the SUMOylated forms of GTF2IRD1. The image shown is a representative Western blot from 4 separate experiments with similar results.

Analysis of the mouse ZMYM5 amino acid sequence revealed no canonical ΨKXD/E SUMOylation consensus motifs, but it contains 2 SIMs in the N-terminal region, which are predicted to have non-covalent SUMO-binding capacity [34]. The ZMYM5-encoding cDNA isolated in the yeast 2-hybrid screen was incomplete and the corresponding peptide lacked the first SIM (^28^EDDDVVFI). However, the second SIM (^88^IVIDDEGD) was intact (Fig. 4B). As ZMYM5 interacts with GTF2IRD1 at or near the site of SUMO ligation and contains 2 SIMs, we predicted that SUMOylation may influence this interaction as well as interactions with other SIM domain-containing proteins such as PIASxβ [17]. HEK293 cells were co-transfected with permutations of plasmid combinations encoding EGFP control, GFP-PIASxβ, GFP-ZMYM5, GTF2IRD1, GTF2IRD1-2KR and HA-SUMO1. Protein extracts were analyzed by co-immunoprecipitation and Western blotting to determine the relative affinities of PIASxβ and ZMYM5 for the non-SUMOylated and SUMOylated forms of GTF2IRD1 (Fig. 5). Compared to the relatively small proportion of SUMOylated GTF2IRD1 detected in the whole cell lysate (Fig. 5A, input lanes), it was observed that PIASxβ preferentially co-immunoprecipitates a much larger fraction of the SUMOylated form of GTF2IRD1 (Fig. 5A). Densitometric analysis of the Western blot revealed that the relative proportion of the SUMOylated form as a percentage of total GTF2IRD1 protein rises from 5% in the input lane to 45% in the co-immunoprecipitated lane, suggesting that SUMOylation increases the level of interaction between these proteins. SUMO-conjugated bands were not detected in the whole cell lysates or in the immunoprecipitated complexes from cells expressing the 2KR mutant protein as expected, although the 2KR protein could still interact with PIASxβ (Fig. 5A). A similar analysis of ZMYM5 interaction with a mixed population of non-SUMOylated and SUMOylated GTF2IRD1 showed the same phenomenon. Levels of SUMOylated GTF2IRD1 were estimated to be 3% in whole cell lysates, but after co-immunoprecipitation with GFP-ZMYM5, the level rose to 16% of the total GTF2IRD1 co-immunoprecipitated (Fig. 5B). These differences were less visually obvious than the PIASxβ co-immunoprecipitations. However, a paired t-test analysis of densitometry data from 5 comparisons in 3 separate experiments determined that a significantly higher proportion of SUMOylated GTF2IRD1 co-immunoprecipitated with GFP-ZMYM5 in comparison to the relative proportion initially present in the whole cell lysate (*P* = 0.0002, n = 5). The GTF2IRD1-2KR mutant co-immunoprecipitated normally with GFP-ZMYM5 (Fig. 5B), suggesting that SUMOylation is not essential for this protein-protein interaction.

### SUMOylation can positively modulate ubiquitination of GTF2IRD1

**Figure 6:**
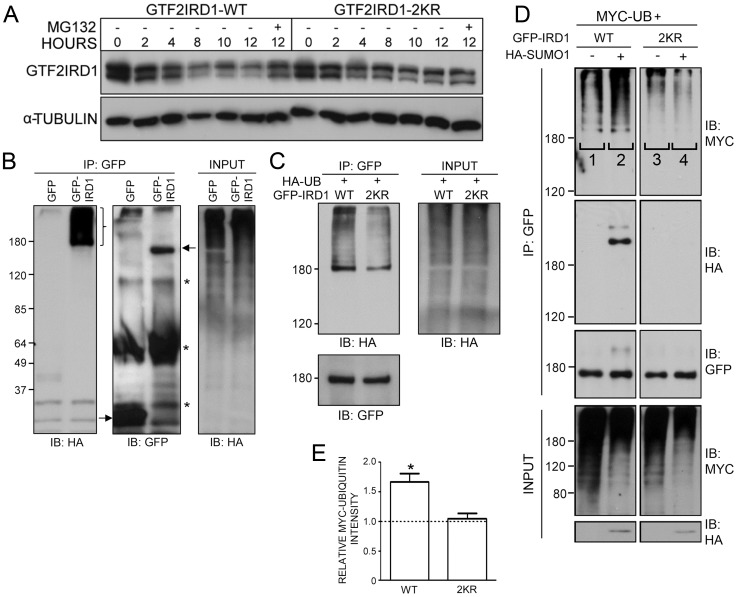
Potential cross-regulation between GTF2IRD1 SUMOylation and the ubiquitin-proteasome pathway. (A) Western blot analysis of GTF2IRD1 degradation rates in HEK293 cells. Cells were transfected with plasmids encoding GTF2IRD1-WT or GTF2IRD1-2KR and extracts were collected at different time points following the addition of cycloheximide alone, or with the proteasome inhibitor MG132. The lower half of the blot was probed with an anti-α-tubulin antibody to control for protein loading. (B) Modification of GTF2IRD1 by ubiquitination shown by western blot analysis of protein extracts from HEK293 cells co-transfected with plasmids encoding HA-Ubiquitin and GFP-GTF2IRD1 or a GFP control. Proteins immunoprecipitated with the anti-GFP antibody (IP) and whole cell extracts (INPUT) were immunoblotted with anti-HA and anti-GFP antibodies. The high molecular weight ladder of bands (}) represents polyubiquitinated GTF2IRD1. Arrows indicate the GFP and GFP-GTF2IRD1 bands and asterisks indicate the IgG heavy and light chains. (C) A similar analysis comparing the ubiquitination levels of GFP-GTF2IRD1 (WT) and the 2KR mutant. The intensity of 2KR ubiquitination is reduced when compared with WT. (D) Western blot analysis of SUMO-assisted GTF2IRD1 ubiquitination. Protein extracts derive from HEK293 cells transfected with plasmids encoding Myc-tagged ubiquitin (MYC-UB) and GFP-GTF2IRD1 (WT) or the 2KR mutant with or without HA-SUMO1. Whole cell extracts (INPUT) or anti-GFP immunoprecipitated proteins (IP) were immunoblotted (IB) with anti-Myc, anti-HA and anti-GFP antibodies. Addition of HA-SUMO increases GTF2IRD1 ubiquitination (compare lanes 1 and 2), but has no effect on GTF2IRD1-2KR (compare lanes 3 and 4). (E) Histogram of densitometric measurements from 3 independent Western blot replicates (a representative example is shown in [D]) to quantify the fold-change increase in the ubiquitination levels of GTF2IRD1 and GTF2IRD1-2KR induced by the presence of exogenous HA-SUMO1 (lanes 2 and 4) over its corresponding control lane without HA-SUMO1 (lanes 1 and 3). A paired t-test analysis showed a statistically significant increase of approximately 1.6 fold in the ubiquitination level of GTF2IRD1 WT (*, *P* = 0.02).

We next investigated whether SUMOylation could play a role in regulating GTF2IRD1 levels and protein turnover via cross-regulation with the ubiquitin-proteasome system. The decay rate of exogenous GTF2IRD1 was analyzed in HEK293 cells following the inhibition of protein biosynthesis using cycloheximide. Western blot analysis of the cell lysates collected at serial time points showed decreasing levels of GTF2IRD1 to approximately 25% of the starting level after 12 hrs (Fig. 6A). Much of this degradation was effectively blocked by the addition of the proteasome inhibitor, MG132, suggesting that a high proportion of the GTF2IRD1 loss can be accounted for by the proteasome. In contrast, an identical analysis on the GTF2IRD1-2KR mutant showed a much slower rate of degradation (Fig. 6A). Densitometric analysis of the levels of GTF2IRD1 and the 2KR mutant proteins remaining after 12 hrs relative to their corresponding MG132-treated controls were estimated to be 65% and 89%, respectively.

These data suggested that GTF2IRD1 is likely to be post-translationally modified by ubiquitin. To test this, HEK293 cells were transfected with plasmids encoding GFP-GTF2IRD1 and HA-Ubiquitin, followed by immunoprecipitation with anti-GFP antibody. Western blot analysis with anti-HA antibody revealed a ladder of high molecular weight bands characteristic of ubiquitinated GFP-GTF2IRD1, which was not detected in the GFP control lane (Fig. 6B). Since the GTF2IRD1-2KR mutant was less susceptible to proteasomal degradation, its level of ubiquitination was compared to wild type GTF2IRD1 in a similar assay. Western blot analysis revealed that ubiquitination of the GFP-GTF2IRD1-2KR mutant was significantly weaker than that of the wild type protein (Fig. 6C).

This observation could be accounted for by hypothesizing that ubiquitin ligation is restricted to the identified SUMOylation lysine residues (K495 and K271) of GTF2IRD1 and thus, mutation of these residues inhibits ubiquitination. However, human GTF2IRD1 contains 60 other lysine residues apart from K495 and K271 and, unlike the dramatic reduction of SUMOylation in the 2KR and K495R mutants of GTF2IRD1, the reduced ubiquitination in GTF2IRD1-2KR was milder, indicating that some of these other lysines act as ubiquitin ligation sites. An alternative proposition is based on recent studies that show how SUMOylation can augment ubiquitin-mediated proteolysis as a result of enhanced interactions with ubiquitin E3 ligases containing SIMs [18], [35]. To test this hypothesis, plasmids encoding GFP-GTF2IRD1 or GFP-GTF2IRD1-2KR were transfected into HEK293 cells, together with plasmids encoding Myc-Ubiquitin and HA-SUMO1. Western blot analysis of cell lysates immunoprecipitated with anti-GFP antibody revealed a significant enhancement of GFP-GTF2IRD1 ubiquitination when HA-SUMO1 was co-transfected, compared with cells that lack exogenous SUMO (Fig. 6D). As shown previously, ubiquitination of the GFP-GTF2IRD1-2KR was considerably reduced, but more importantly, the addition of exogenous HA-SUMO1 failed to augment the levels of GFP-GTF2IRD1-2KR ubiquitination (Fig. 6D). Quantification of these ubiquitination levels was achieved by densitometric measurements of the ubiquitin ladder intensities from 3 independent experiments (Fig. 6E). Addition of HA-SUMO1 augmented an approximate 1.6 fold increase in ubiquitination of GTF2IRD1 (*P* = 0.02), but had no significant impact on the ubiquitination of GTF2IRD1-2KR, demonstrating that this effect can be attributed to GTF2IRD1 SUMOylation and not to an indirect mechanism.

## Discussion

In this study, we have shown that human GTF2IRD1 can be SUMOylated through the combined action of the E2 conjugating enzyme UBC9 and the E3 ligase PIASxβ. The importance of SUMOylation in GTF2IRD1 function can be inferred from the level of conservation found at and around lysine 495 where the SUMO moiety is attached (SUMO1) and the C-terminal region where PIASxβ binds. Alignment of amino acid sequence from the GTF2IRD1 orthologs present in human, mouse, frog and fish showed perfect conservation of the SUMO1 motif (^494^IKIE^497^), in comparison to the less well conserved SUMO2 motif (^270^LKQE^273^). The residues surrounding the consensus sequence of the SUMO1 site also show higher conservation, indicating resistance to evolutionary divergence due to the functional constraint of SUMOylation. Similarly, sequence conservation in the 27 amino acids at the C-terminus, where PIASxβ was shown to bind is also very high (22 out of 27 residues or 81% identity between fugu and human; data not shown).

A novel interaction with ZMYM5, a putative member of the HDAC complex, was also identified in our study. ZMYM5, also known as ZNF237, belongs to a family of proteins (ZMYM1–5) that share specific MYM-type zinc finger domains (Cys-X_2_-Cys-X_19–22_-Cys-X_3_-Cys-X_13–19_-Cys-X_2_-Cys-X_19–25_-Cys-X_3_-Cys). Although the MYM domains are thought to mediate protein-protein interaction [36], our mapping analysis showed that the MYM domains are not responsible for ZMYM5 interaction with GTF2IRD1, but rather through a conserved region which has not yet been classified as a known motif. ZMYM5 shares extensive homology with the N-terminal region of ZMYM2 and the genes encoding these proteins are located adjacent to each other on chromosome 13, suggesting that they have arisen by duplication and divergence of a common ancestral gene [33]. It is intriguing to note that ZMYM2 and ZMYM3 together with TFII-I, the close relative of GTF2IRD1, have been co-purified within a HDAC co-repressor complex from extracts of HeLa cells [37]. Involvement of TFII-I and GTF2IRD1 with the HDAC complex has previously been reported through the identification of TFII-I in a purified HDAC3 immuno-complex [38], [39] and the binding of recombinant GTF2IRD1 to a complex immunoprecipitated using an anti-HDAC3 antibody [40]. The ZMYM proteins are thought to act as adaptor proteins mediating protein-protein interactions through their MYM-type zinc fingers [41]. Therefore, we propose that ZMYM2, 3 and 5 mediate the interaction of GTF2IRD1 and TFII-I with HDAC co-repressor complexes.

Our finding that SUMOylation enhances GTF2IRD1 interaction with ZMYM5 adds to the accumulating evidence that the SUMO mechanism engages with multiple corepressors to regulate chromatin structure and thus transcription [14]. Both ZMYM5 and ZMYM2 have been shown to interact non-covalently with SUMO1 via a SUMO-interacting motif (SIM) [34] and SIM-mediated binding in ZMYM2 was shown to be important for its interaction with SUMOylated HDAC1 [41]. SIMs allow proteins to interact with the SUMO motif in a non-covalent way and hence couple SUMOylated proteins to downstream regulatory events [16], [34]. SUMOylation of GTF2IRD1 also enhances its affinity for PIASxβ, which could form a positive feedback loop that maintains the interaction with the E3 SUMO ligase and increase the likelihood of further SUMOylation. Alternatively, additional reasons for a sustained interaction between between SUMOylated GTF2IRD1 and PIASxβ are possible. PIAS proteins have been shown to re-localize protein partners to different regions of the nucleus and are implicated in the organisation of chromatin structure and interactions with nuclear matrix attachment regions [42].

Our study also showed for the first time that GTF2IRD1 is modified by ubiquitination, a process largely responsible for targeting proteins for degradation via the proteasome machinery. Ubiquitin conjugation shares many similarities with SUMOylation, but one of the exceptions is its essential requirement for the involvement of an E3 ligase. Ubiquitin E3 ligases can be broadly classified as HECT (homologous with E6-associated protein C-terminus) domain-containing E3s and RING domain-containing E3s [43]. A novel family of RING domain ubiquitin ligases, called SUMO-targeted ubiquitin ligases (STUbLs), were found to recognize SUMOylated substrates [18] and use SIMs within their structure to bind the SUMO moiety of other proteins in a non-covalent manner [35]. In this study, we showed evidence of a possible cross-regulation of GTF2IRD1 by SUMO and ubiquitin. Since SUMO increases ubiquitination of GTF2IRD1 in our assay system, we hypothesize that SUMOylation facilitates or enhances the binding of a SIM-containing ubiquitin E3 ligase in a similar manner to PIASxβ and ZMYM5.

WBS is a disease that is caused by haploinsufficiency of dosage-sensitive genes that fall within the hemizygous deletion. The previously demonstrated direct negative auto-regulation of *Gtf2ird1* transcript levels [28] and the post-translational regulation shown in this paper indicate that GTF2IRD1 has evolved mechanisms that ensure tight regulation of activity and abundance. This evolutionary adaptation implies that cells are sensitive to the dosage of GTF2IRD1 and it is not difficult to imagine how hemizygosity of *GTF2IRD1* in WBS would disrupt this finely controlled balance resulting in deleterious consequences.

## Supporting Information

Table S1 **Names and sequence of the oligonucleotides synthesized for all experiments.**
(PDF)
