# Supplementary material for: SUMOylation of GTF2IRD1 Regulates Protein Partner Interactions and Ubiquitin-Mediated Degradation
Source: PLoS One. 2012 Nov 8;7(11):e49283. doi: 10.1371/journal.pone.0049283 (PMC3493543; doi:10.1371/journal.pone.0049283)
Supplement: Table S1 — Names and sequence of the oligonucleotides synthesized for all experiments. (PDF) [file pone.0049283.s001.pdf]

SUPPLEMENTARY MATERIAL

Name	Sequence (5' to 3')
**HMER1F**	ATGAATTCACCATGGCCTTGCTGGGTAGCGCTGTGACGTCCCC
**HMDCDSR**	ATCTCGAGCTAGTAATTAAGAGGTCCCGGGAGCTGCACGTTCAGG
**LZ-forward**	TTGAATTCAACGGCTGCGGACCCGAC
**LZ-reverse**	TTCTCGAGATCCTTCCACGGGGGCCC
**RD1-forward**	TTGAATTCGGCTCAGATGTGTACCTTCTG
**RD1-reverse**	TTCTCGAGCCGCCCGCCATCCTCAAGTGG
**RD2-forward**	TTGAATTCACCGAGGACATCAACACGCTC
**RD2-reverse**	TTGAATTCACCGAGGACATCAACACGCTC
**RD3-forward**	TTGAATTCGGTGACGTGATCCGGCCCCTG
**RD3-reverse**	TTCTGGAGTCCCTCAGTGAGCAGCTCGGG
**RD4-forward**	TTCTGGAGTCCCTCAGTGAGCAGCTCGGG
**RD4-reverse**	TTCTCGAGTGGGATGAGTCCTTGGAAAGG
**RD5-forward**	TTGAATTCCTCGGGGAGAAGGTGATCCTG
**RD5-reverse**	TTCTGGAGTTCTGCAAAGGGTTGGAGCTG
**Sumo1-forward**	TTCTGGAGTTCTGCAAAGGGTTGGAGCTG
**Sumo1-reverse**	TTGTCGACCTCACTCAGACCTTTGTC
**Sumo2-forward**	TTGAATTCGGGTCACGGGACTGTGGCCTG
**Sumo2-reverse**	TTCTCGAGACCAGGCCCAGTGGGCTTCTG
**Tr-forward**	ATGAATTCACCATGGCCTTGCTGGGTAAGCGCTGTGACGTCCCC
**Tr1-reverse**	ATGTCGACCTAGTTGGCCGATGCCACTGAATC
**Tr2-reverse**	ATGTCGACCTAATTTCCTTCCGAGACCCGC
**Tr3-reverse**	ATGTCGACCTACTTTGGGATGAGTCCTTGG
**PIASxF**	GTCTCGAGCCATGGCGGATTTCGAGGAGTTG
**PIASxR**	ATGAATTCCTTTAGTCCAAAGAGATGATGT
**HMDCDSF**	ATAAGCTTACCATGGCCTTGCTGGGTAAGCGCTGTGACGTCCCC
**HMDCDSR**	ATCTCGAGCTAGTAATTAAGAGGTCCCGGGAGCTGCACGTTCAGG
**UBC9F**	TAGGATCCATGTCGGGGATCGCCCTCAG
**UBC9R**	TAGAATTCTTATGAGGGGGCAAACTTCTTC
**ZMYM5F**	TTCTCGAGACATGGAAGCTCATCTCGCAG
**ZMYM5R**	TTCCCGGGTACCTATGTGTACAGTAGCTGCACAC
**ZMYM5Tr99**	ATCTCGAGATTTGTGTCTGTGTCCC
**ZMYM5Tr147**	ATCTCGAGCCTACCGGGATTAAAAG
**ZMYM5Tr233**	ATCTCGAGAGCTGATCCTTTTCGCTG
**ZMYM5Tr325**	ATCTCGAGTGTTATACTGTTAACAC

Supplementary Table S1. Names and sequence of the oligonucleotides synthesized for all experiments.
